# Anæsthetics Medico-Legally Considered

**Published:** 1882-03

**Authors:** J. G. Johnson

**Affiliations:** Brooklyn, New York


### THE DENTAL REGISTER.

VOL. XXXVI.] MARCH, 1882. [No. 3.

### ANÆSTHETICS MEDICO-LEGALLY CONSIDERED.

BY J. G. JOHNSON, M. D.
Brooklyn, New York.

Mankind has in all ages and in all climes sought relief from pain.

What reader of the Odyssey cannot call to mind the beautiful Helen driving away sad memories from the minds of her husband and friends by making them drink of the wine into which the sweet nepenthe had been cast—that nepenthe that delivered men from grief and wrath, and caused oblivion to every ill?

The effects of alcohol in stupefying and overpowering the nervous system was known as far back as we can trace the history of civilization. In the Bible we read of the daughters of Lot administering wine to their father and entering in unto him that the human race might not die out. The Chinese are known as far back as their history can be traced to have used hashesh or Indian hemp to relieve pain of acupuncture.

Inhalation of narcotics was known to the ancients. Herodotus relates that the Scythians were accustomed to produce intoxication by inhaling the vapor of a certain kind of hemp. The fertile plains of India produced the soporiferous poppy from which opium was extracted, whose power of relief is so great that it has justly been called "the great gift of the Gods to men."

Among the Egyptians was an art of producing sleep by inhalation.

Pliny describes a mineral, brought from Memphis, which, when pulverized and mixed with sour wine, applied to a wound, would destroy pain.

Baron Larry, after the battle of Eylau, found in the wounded who required amputations a remarkable insensibility owing to the intense cold, this being the first use of cold as an anæsthetic.

Of all drugs known to the ancients, mandragora wine undoubtedly was the most potent and efficient. Apulius states that half an ounce of this preparation would render the patient insensible to even the pain of amputation.

The wine mingled with myrrh offered by the women to our Savior on the cross was undoubtedly, mandragora, for it was a common practice for kind-hearted women to alleviate the horrible agonies of those being crucified by the administration of this pain-defying drug.

Dr. B. W. Richardson, of London, proved that this was true. He procured a specimen of mandragora root, from which, though five centuries old, he made mandragora wine, and administered it to criminals, and found that it was a narcotic having precisely the properties claimed for it. He found that in animals it would produce the sleep of Juliet, not for forty hours, which must be regarded as a poetic license on Shakespeare's part, but for four hours, as described by Dioscorides. Who could have written Friar Lawrence's description of the wine that Juliet is to drink unless he had seen its effects?

> "Through all thy veins shall run
> A cold and drowsy humor, which shall seize
> Each vital spirit; for no pulse shall keep
> His natural progress, but surcease to beat;
> No warmth, no breath, shall testify thou livest;
> The roses in thy lips and cheeks shall fade
> To paly ashes; thy eyes' windows fall
> Like death when he shuts up the day of life:
> Each part deprived of supple government
> Shall stiff, and stark, and cold, appear like death;
> And in this borrowed likeness of shrunk death
> Thou shalt remain full two and forty hours,
> And then awake as from a pleasant sleep."

But to our country and century is the world indebted for the discovery and application of anæsthetics for the purpose of rendering persons insensible under surgical operations. If America had contributed nothing more than this to the stock of human happiness, the world would owe her an everlasting debt of gratitude. The name of Morton, of Boston, will descend to posterity as a benefactor of the human race; the benefaction he has conferred on suffering humanity in the relief of pain being as great a boon as those conferred by those other Americans, Fulton, who first applied the steam engine to navigation of vessels, and Whitney, who invented the cotton gin, have been to the material prosperity of the world.

No one can form, even at the present day, a just estimate of the true value of the various anæsthetics, or express in words their wonderful and extended application to human suffering. To the surgeon it gives the means of operating and saving life in grave cases, when before the discovery of anæsthetics the shock rendered death inevitable. It affords the surgeon the means of manipulating the broken bones and injured parts with facility, when without it the patient would be writhing in agony. With anæsthetics a correct diagnosis can be made in the most obscure injuries and painful diseases. To the woman in the pangs of labor it brings sweet relief to the primeval curse, and she comes to her confinement with the happy consciousness that she will not suffer as her mother did. In the removal of large tumors it affords relief to the shock—the most dreadful result of all operations. It renders the eye insensible, so that it can be touched with impunity, and the most delicate operations performed on this sensitive organ without pain and with far less risk.

While thus suffering humanity is benefited incalculably, still the surgeon should remember every time he takes an anæsthetic in his hand that he is bringing his patient far down on the road to death, where the slightest error on his part may prevent return to life.

Nor is this a blessing unmixed with evil. Though it is to American dentists that the world is indebted for anæsthetics, yet the dentists themselves have often had to suffer an agony which has made some of them wish their profession had never conferred this boon on humanity.

The surgeon or dentist should never administer chloroform to a female patient unless some other female is present and stays in the room the whole time, from the commencement of the inhalation to the restoration to full consciousness of the patient. Although women more often than men have their sufferings alleviated by anæsthetics, they have too often rewarded the dentist with charges of the most alarming character, and he has been dragged into the criminal courts to answer.

Scarcely had anæsthetics been introduced into the profession when, in 1847, a dentist in Paris was condemned to punishment on a criminal charge brought by a female patient to whom he administered ether for the extraction of a tooth.

The case of Dr. Beale, the Philadelphia dentist, is thoroughly known to both the legal and medical professions, and is fully reported in *Wharton & Stille*, so that it is only necessary to allude to it. There are so many other cases scattered through our medical literature that are not so generally known that I will quote a few of them.

In the Philadelphia *Medical Times*, December 22, 1877, is the following case, taken from English authorities:

"A case of the utmost importance to the whole profession, not in Great Britain only, but everywhere, was tried before Mr. Justice Hawkins, of Northampton, on the 9th of November. It was a charge against a surgeon's assistant of criminal assault—of rape upon a patient when under the influence of chloroform. If there is a dastardly crime, it is to take advantage of a woman's helpless condition to violate her person, and so the magistrate thought, who sent the accused to jail on the 14th of September, declining to hear anything in his favor, and resolutely refusing to accept bail. The charge was that a married woman named Child went to the surgery of her family medical attendant to have her teeth operated on. She had been there a day or two before, but the attempt to put her under chloroform then failed. A second attempt was rather more successful. She evidently had some peculiarities or idiosyncrasies in relation to chloroform, for he gave it for an hour, and yet she was never sufficiently under its influence to permit the operation being performed. She was accompanied by a friend—a Miss Fellows. At the end of the hour, Miss Fellows went out of the room and saw Mr. Child. In a quarter of an hour Miss Fellows returned. The prosecutor maintained that on Miss Fellows' return she was quite conscious but unable to speak.

Finding that it was impossible to perform the operation, the accused accompanied the prosecutrix and her friend home. So far, Mrs. Child had been unable to speak, but shortly after the accused left the house, she complained to her husband that he had taken advantage of the absence of Miss Fellows to assault her criminally. Next day when the accused called, he was told what she had said, and he replied that she was laboring under a delusion. Under cross-examination, Mrs. Child said that she told the accused that if he would admit the offence and quit the town (Birmingham) she would forgive him. This the accused declined to do, denying that he had committed any offence. He was then given into custody.

The prosecutrix stated that the offence was perpetrated immediately after Miss Fellows left the room; that the prisoner went upon his knees and assaulted her. Miss Fellows stated that on her return she found Mrs. Child in precisely the same position in the chair which she occupied when she went out of the room. Such were the facts of the case.

It was quite clear that there had been an assault committed or the woman was under the influence of a very pronounced delusion. The whole of the accused's conduct was in favor of the latter hypothesis, but in such a matter, where no third person is present, the statement of one of the two parties concerned must be taken. When a woman whose character was apparently without blemish (for in cross-examination no attempt was made to call her reputation into question,) makes a definite charge against a man of assaulting her, under circumstances which permitted of such an assault, the law could only send the case to a jury. In the mean time the unfortunate surgeon's assistant was sent to prison.

When the case came to be tried, a large number of medical men of repute came forward voluntarily, paid the accused's defence, and did this gratuitously. The chief witness for the defence was Dr. B. W. Richardson, whose celebrity is world-wide. As is well known, Dr. Richardson has studied anæsthetics very carefully, and for many years. He stated there were four stages or degrees in which chloroform operated. The first stage was that in which consciousness was not lost. There was resistance, and a desire for air. In the second, consciousness is lost, but the operation is impossible, the patient screaming, often without provocation. The third stage was that of complete unconsciousness, and when all rigidity is lost. This is the stage that permits the operation. In his opinion the patient was in the second stage, the third never having been reached. He stated that in his own experience he had known persons to have delusions as to what had taken place during that time. He related a number of cases, and stated that the fact of such delusions being induced by chloroform, was one of the earliest objections raised to its adoption.

He related one case where the patient, a female, was being operated upon by a dentist, and alleged that the dentist criminally assaulted her; and this she persisted in, though her own father and mother, Dr. Richardson, and the dentist's assistant were all present throughout the whole time. She persisted in that conviction long after the effects of the chloroform passed away, and Dr. Richardson said she was probably of that belief still. The evidence of Dr. Richardson was corroborated by the experience of Dr. Hawksby, of London, and by Dr. Saundby and Mr. I. F. West, of Birmingham. The judge asked the jury if it was necessary to sum up, and they replied that it was unnecessary, they were already agreed on a verdict of acquittal. Mr. Justice Hawkins pointed out that such a verdict would not be the slightest imputation on the absolute sincerity of the prosecutrix, who, no doubt, firmly believed every word she had said. He then congratulated the accused upon having had an opportunity of fully vindicating himself from the charge preferred, and said that the verdict of acquittal did not mean that there was insufficient evidence but the accused was entirely clear of any imputation in regard to the charge preferred against him. There could be no doubt that the prosecutrix labored under a delusion. The accused was then discharged from custody, having been in prison two months for no offense.

It was not merely that this unfortunate man was imprisoned two months for an imaginary offence, but that any man who is present when a woman is being put under chloroform is liable to have the same charge preferred against him that gives this case its gravity and importance. Such being the case, it becomes necessary that a little more should be known amidst the profession, as well as the laity, as to the occurrence of erotic sensations in women. This subject is not a pleasant one, but that is no reason why it should not be investigated. If it is a fact—and there is no doubt about this—that women when being put under chloroform, are liable to those erotic sensations which they experience from sexual intercourse, the sooner the fact is generally known the better. It is just the mystery that surrounds such facts that permits such a monstrous hardship as that mentioned above to be a possibility at all. Of course, it is obvious enough to any one that it is a delicate matter to inquire into the subjective sensations of woman; but if these subjective sensations take the practical form of a charge of rape, two months in jail, and a trial by jury, they pass from the domain of sentiment and enter that of stern reality."

In the *Medical and Surgical Reporter* of January 12, 1877, Dr. N. L. Folsom of Portsmouth, N. H., relates the following case:

"In 1854, a clergyman's sister came to my office for the purpose of taking ether and having a tooth extracted, and brought her brother's wife with her. I began to administer the ether to the patient, and while renewing it she got away from me and seemed alarmed and offended. I did not attempt to compel her to breathe any more ether, but urged her to take it, and so also did her brother's wife; but she would take no more. She had the impression (so her brother told me,) that I attempted to violate her, and that his wife assisted me. It was a long time afterwards before she would fully give up that she was mistaken in the matter."

In the *Boston Medical and Surgical Journal* for November, 1858, page 287, is the account of the trial of a dentist in Montreal for an alleged attempt to commit rape upon one of his patients to whom he had given chloroform. "One of the witnesses for the defence testified that his own wife was fully impressed with the belief that she, too, had been violated by the prisoner, under the influence of chloroform; and she persisted in this delusion, notwithstanding that her husband was present during the whole period of anæsthesia. In spite of this and other testimony, the jury brought in a verdict of an attempt to commit rape, though they had the grace to join with it a recommendation to mercy."

*Wharton & Stille* relate a case in which, while enlarged nymphæ were being removed, the woman went unconsciously through the movements attendant on sexual orgasm, in the presence of numerous bystanders.

Corroborate evidence of this stimulating effect of the various anæsthetics upon the sexual senses of females can be collected from the experience of almost every gynæcologyst; and the legal profession should be made thoroughly aware that this is a fact; and, as *Wharton & Stille* well say, "Testimony of witnesses as to what took place when they were under the influence of chloroform should be subjected to the same tests as those applied to insane witnesses. Such testimony can not be safely excluded; but there should be always in such cases proof of the *corpus delicti aliunde.*"

Anstie, a celebrated English authority, has added immensely to the knowledge of the profession on this subject. He experimented on various animals, carrying the chloroform narcosis to death, and he invariably found in every animal that the ano-genital region was the last to give up its sensitiveness—that by irritating the ano-genital region he could produce a response when all other parts were so thoroughly narcotized that death was impending.

Anstie's precise language at the conclusion of his experiments is of interest: "The first effect on sensation which is noticeable is the removal or mitigation of any pain from which the patient suffers. This is often effected by one or two inspirations only. Evidence of paralysis of skin sensibility, on the other hand, could only be obtained in four patients during the first minute, and in the lower limbs. Before the end of the second minute, however, there was considerable paralysis of the whole skin surface in forty-seven out of fifty patients. The conjunctiva always retained sensibility later than the skin, with certain exceptions, presently to be noticed. In the great majority of instances, however, it was rendered insensitive by the time the contraction of the pupil was well marked (third stage). Certain portions of the skin and subcutaneous tissue, however, retained their sensibility with extraordinary tenacity; these are the matrix of the great toe nail, the margin of the anus, and the whole of the skin of the organs of generation. It is impossible to obliterate their sensibility without pushing chloroformization to a degree which greatly surpasses that required for ordinary purposes. This observation is confirmed by my experience with animals, and its importance cannot be too highly estimated, for it explains the frequency with which death has happened in the course of anæsthesia, induced for the performance of operations for phymosis, evulsion of the toe nail, hemorrhoids, etc. All kinds of fanciful reasons have been given for the fatality of chloroform in such trifling operations, but there is no doubt in my mind that this is the true one."—*Stimulants and Narcotics.* By Francis E. Anstie, M.D., M.R.C.P. Philadelphia: Lindsay & Blackiston, 1865.

With this fact demonstrated, as it has frequently been by others since he brought it to the notice of the profession, the explanation of these grave charges made by females of respectability becomes easily enough understood. Those parts conveying sensations after all other parts have had the sensibility overpowered, the pressure of the clothing against the parts, stimulating them as she is struggling under the influence of chloroform, and her being held, naturally conveys to her the idea of something wrong, not knowing beforehand that the effect of chloroform will make her struggle and resist, and that she may be forcibly held down—when it does occur, and this sexual stimulation also occurs, the facts, to her mind, are overwhelming, that the restraint was for the purpose of sexual gratification; and she testifies as she honestly believes.

I would call the attention of the legal profession to this discovery of Mr. Anstie as the result of his experimentation on the animal creation. If vivisection and experiments on the brute creation had added nothing more than this to the stock of medical knowledge, that one fact alone would be worth more than the lives of all the mongrel dogs with which our large cities are infested; and the next time Mr. Bergh attempts to prevent, by legislative enactment, the medical profession from experimenting on the dumb creation, this and corresponding facts should be brought to the notice of our legislators.

With the present knowledge of the profession of the facts that chloroform does stimulate the sexual functions of the female, and that the ano-genital region retains the power of conveying sensations after sensibility is lost in other parts, let us revert to the conviction of Dr. Beale, the Philadelphia dentist, who was sentenced to ten years' imprisonment upon the unsupported statements of the female herself. There was no physical examination of her person, either immediately after the alleged assault or at any time previous to the trial. According to her own statement, she was accompanied to the dentist's door by her accepted lover, who left her there with a kiss upon her lips, and naturally with her sexual feelings more or less aroused from his presence and contact. In addition to this, her menstrual flow was due that very day. In this stage of sexual excitability she was closeted with an agreeable gentleman who had jestingly, some time before, talked of making her his wife. In this excitable condition she inhales ether, and what more natural than that its stimulating effect upon her at that time should cause her to honestly believe just what she swore to—that her ruin was effected by the dentist, and she was powerless to prevent it? And yet her whole statement contradicts that whole theory; for if she had been a virgin, as claimed, this forcible connection would have ruptured the hymen, *and there would have been an immediate flow* and stain of her linen, and that soreness of the parts which so clearly tells of the wrong committed. After the occurrence she takes a walk; goes to see a friend. Her periods do not come on till the after part of the day—their usual time—and she does not mention the occurrence till evening, and then to a lady friend. What are the probabilities from her own statement? Would she not have gone home to her mother, or gone to her lover, and told it at once? With charges as injurious as this to the physician, breaking him up in business, with his reputation destroyed, with the story to follow him wherever he goes, the statement of the woman should have the strongest corroborate proof, both from the physical examination of her person and linen immediately afterward, and other extrinsic evidence.

The dentist and physician should also protect themselves by absolutely refusing to administer either chloroform or ether unless some lady is present and in the room the whole time of the administration and till full return to consciousness.

Another medico-legal point of interest to the physician, and also to the lawyer, is the question—what would be the effect on the prisoner's case of a patient dying from the effect of chloroform administered for surgical purposes? To make the question plain: suppose that the surgeons should have found the bullet in President Garfield's case, and after consultation of careful, skilled surgeons, it should have been determined to put him under the influence of chloroform to remove the bullet, and he had died from the chloroform before the operation had been commenced, would it relieve Guiteau from the charge of murder? Under the old rule of law, unskillful treatment on the part of the attending surgeon did not relieve, even if it could be shown that the treatment was unskillful and expedited the death. It was held, and justly, too, that the prisoner was responsible for the result, because if it had not been for the wounding the attendance would not have been necessary. But here is a different case: The physician administers a well-known poison. Hundreds are known to have died from the effects of this poison. Those have died from the effects of it who were carefully examined beforehand to see if it was safe to administer it in their case. Physicians have died from it when carefully administered by other physicians, in whose skill they had confidence. Cases also are on record of deaths from chloroform in our large hospitals, where the ablest of our profession have been in attendance, and they have had the honesty and manliness to come forward and state that after a careful post-mortem examination they could find no cause that would explain the reason why the chloroform produced the death. Death has occured from Squibs' chloroform, from Powers & Weightman's, from Duncan & Lockhardt's, manufacturers of the highest repute.

Now, the patient dies from the effects of a well-known poison, administered by another party—with good intent, it is true, but, nevertheless, he dies from a poison—he does not die from the wound. If he does not die from the wound, then there is no murder. If there is no murder, then there is no murderer; and the prisoner can only be convicted of a felonious assault. When this question has been first put to my legal friends, their answer has invariably been according to the old ruling of the courts. After the matter has been laid before them they admit that it is a close question; that it must be shown absolutely that the wound was a fatal one. The prisoner is entitled to the benefit of every doubt. With the enormous number of recoveries that have taken place in doubtful cases, no surgeon could swear positively but what the patient might have recovered had not the chloroform been administered, and then the case would have been one of feloneous assault. I have been unable to find any case where the courts have passed upon this question. The last edition of *Taylor's Medical Jurisprudence* has the following, bearing on it:

"In a case of this kind, what becomes of the responsibility of the party who inflicted the original wound? No decision, so far as I know, has ever been given on this point. Was the administration of the chloroform vapor, in a professional view, a necessary part of the treatment? Was it skillfully and properly administered? Could the deceased condition of the heart, which rendered the effects of the vapor more fatal than usual, have been detected by the operator so as to show the impropriety of administering it in this case? These questions should receive satisfactory answers before the aggressor is rendered responsible for death under these peculiar circumstances."

Should the prisoner be acquitted by a jury, in one of these cases would an action lie against a physician who had administered the chloroform, and have been the unwitting cause of death? This is a question of great importance to surgeons connected with our large institutions, where injuries and woundings of every kind are continually brought in, and where, to save life, a surgeon must often act promptly where he knows nothing previously of the patient, and perhaps can learn little more, either from the exhausted or intoxicated condition of the patient, and yet must give his immediate and best attention; if a physician, under these circumstances, does that which seems best to his enlightened judgment, is he responsible should the result prove unfortunate?

The tendency to draw more strictly, the line of responsibility is shown each year. Should a patient die from chloroform inhaled in a sitting position in a dentist's chair, it could no longer be urged in behalf of the surgeon whose patient had been chloroformed out of existence, as it was successfully argued in behalf of the young Parisian surgeon in 1853, who had been imprisoned for the death of a patient under chloroform, on whom he was operating without assistance, that there were no fixed rules for the administration of chloroform. The English Chloroform Committee appointed by the Royal Medico-Chirurgical Society laid down, in 1864, the rule that anæsthetics should always be given in the recumbent position, and never in the erect position. The reason of this rule is evident. In natural respiration the rising and falling of the ribs is produced by the intercostal muscles, and the respiration is called thoracic. As the patient comes under the influence of the anæsthetic these intercostal muscles become paralyzed and cease their action. The respiration is then kept up by the action of the diaphragm, or abdominal respiration. Those who have seen much of the patients under the influence of anæsthetics in our large hospitals, must have noticed how quickly a patient stopped breathing at this stage, if an assistant pressed against the abdomen, to watch the operation, or to pass an instrument. Now, as soon as the patient comes fully under the influence of an anæsthetic, she slips down in the dentist's chair. The weight of the upper portion of the body is compressing the abdomen, preventing the diaphragm from acting.

I think, with the present knowledge of anæsthetics, that a surgeon who should administer chloroform to a patient in the erect position in the dentist's chair, with her clothes tight around her waist, and the patient should die, he would justly be held for manslaughter. During the early ages of anæsthetics, the knowledge of the profession was only experimental. That age has passed. The most distinguished men in the profession, as long ago as 1864, published this rule and the reason for it. Subsequent experimentation has demonstrated the justice of it. It has been adopted by all our modern writers on the subject. The courts have held, over and over again, that a physician must practice according to the well-known rules of his profession, and if he departs from them it is at his peril. Now, if he has read any of the medical authorities issued in the last fifteen years, he must have seen this warning. If he neglects it, and a life is lost by his neglect, what escape is there from the charge of manslaughter?

Next to deaths in the dentist's chair comes the frequency of deaths from operations for piles and other operations of this kind about the rectum. When we recall the fact of the ano-genital region being the last to give up its sensitiveness we can understand this result, because the boundary line between danger and death is so slight, that before the anus ceases to respond the patient is across the line of fatal narcosis. Again, the patient is rolled over very frequently for the surgeon's benefit, and thus there is danger of interfering with the abdominal respiration.

Anæsthetics should never be used for these cases so as to produce full loss of sensibility. Anæsthetics should not be used at all unless the patient is very nervous, and only enough to blunt the susceptibilities of the parts. With the knowledge of this peculiarity of the ano-genital region given the profession by Anstie, and so thoroughly the property of the profession, failure to observe these precautions will eventuate in the surgeon's being held on a serious charge should death follow, unless he could show that the operation was of sufficient gravity to call for this unusually dangerous use of anæsthetics; that it was not merely his individual opinion, but that it was the joint opinion of a consultation; that the anæsthetic was administered by a person skilled in the business, and not by a medical student; and that the person who administered the anæsthetic had no other part of the operation to attend to to distract his attention from the effect the anæsthetic was having on his patient.

This is a point the surgeon should well consider, for it is an established principle of law that a physician or surgeon in the performance of his professional duties is liable for injuries resulting from his want of ordinary diligence, skill, and care.—Landon *vs.* Carter, 9 Conn., 209. Already, in England, according to the last edition of *Taylor's Medical Jurisprudence*, cases of this kind have been brought before the courts—suits for damages for deaths from chloroform—and although they broke down from a failure of the proof, still the courts held that the same principle of law was applicable to these cases as to other civil suits against physicians.

Another question of interest is whether it is justifiable to use chloroform against the person's will to detect fraud. During the war soldiers would imitate the curvature of the spine or stiffness of a joint, or some other disease, as deafness. This malingering could always be detected by putting the patient under the effects of chloroform. The same trick is frequently done now by persons arrested under some criminal charge, who often simulate deafness or some other defect to conceal their identity. It is an interesting fact that no matter how much you may inculcate upon a patient that he shall not speak, he will invariably answer when coming from under the effects of chloroform. The stiff knee, apparently permanently anchylosed, will bend, and the curved spine, that they have learned to simulate so well that it will remain curved in sleep, becomes straight; and there is not a simulated disease but can be discovered by chloroform.

Has the surgeon any right to make use of this means? With the arrested party, I think decidedly not. Every party is to be presumed innocent till proved guilty, and the law will not compel a person to criminate himself. It would seem that the old opinion holds that a prisoner's person was sacred, and that evidence obtained in that way would be ruled out by the courts if obtained against the will of the party. So far has this principle been carried that the Court of Appeals has decided that a judge cannot even make a prisoner stand up in his seat for the purpose of having a witness on the stand identify him. As for enlisted persons, the army officer would probably enforce it, claiming *inter arma leges silent;* and besides, the party has voluntarily placed his life under the orders of his superior by enlisting, and absolute obedience is the law of the service known to the recruit beforehand. The question becomes interesting to the medical expert who may be sent by order of court to make an examination, under an anæsthetic, to ascertain whether the claim is honest or not. He is expected to testify to the truth. How can he testify if he is not allowed to obtain his facts?

This right to use chloroform against the will of the party upon whom it is to be used, becomes an interesting question, and has been brought before the courts. My invariable custom, in these cases, is to request that the patient may be placed under the effect of an anæsthetic by her attending surgeon, that I may examine it in his presence, and then leave with them the responsibility of the refusal. If the injury is feigned, their excuses will injure them usually as much as an examination of the case would--and you have got the information needed without putting yourself in the position of a trespasser, in endeavoring to administer anæsthetics against the will of the party.

The question that was so exhaustively discussed by a former president of this society, Stephen Rogers, M.D., has been renewed recently in our courts.

Dr. J. V. Quimby, of Jersey City, was called as a witness in the noted case of the murder of the policeman, Richard Smith, by his wife's paramour. She claimed that she was asleep in bed with her husband when the murder was committed, hence the blood on her under-clothing. She was tried as a *particeps criminis,* the State holding her claim that she was chloroformed in her sleep to be an impossibility. Dr. Quimby was called as a witness. He knew nothing of the possibility of administering chloroform successfully while asleep. He tried it, and found that he successfully chloroformed three persons, one a man, the other two being boys, one of ten, the other thirteen years of age. He took about seven minutes to chloroform the man. *Transactions of American Medical Association*, 1880.

The experiment has frequently been tried by experts since Dr. Rogers' paper, and it has been found that with children it can be done by a skilled hand after a little practice; but with adults it is a matter of great difficulty, and unless the person who uses the chloroform is an expert, it is an utter impossibility.

In a trial for an attempt at robbery by the use of chloroform, at New Bloomfield, Pa., the possibility of anæsthetizing a sleeping person was discussed in court by Dr. F. F. Maury and B. Howard Rand, M.D., professor of chemistry and lecturer on medical jurisprudence in Jefferson Medical College. Dr. Maury stated (*Wharton & Stille,*) that he had experimented on six sleeping persons. All resisted, more or less. Two woke up immediately, and one remarked, "You are trying to give me something." He administered it to a child four days old. It offered resistance. With all his experience he could not administer it to four persons. Professor Rand confirmed Dr. Maury's testimony.

The question that anæsthetics should be used has been much discussed by the profession, and there is a wide difference of opinion. In England, and on the Continent, chloroform holds almost entire sway. They claim that the deaths result from ignorance and carelessness in its use; that if the proper amount of atmospheric air is administered with it, it is practically free from danger; that not more than three and one-half per cent. of chloroform should be allowed; that if the party who is to administer the chloroform attends to that and that only, and knows his business, and watches the respiration and face, and, should they show any danger, pull the tongue violently forward with a pair of rat-toothed forceps, so as to start up the respiration immediately, the danger is averted. They point to the fact that during the entire Crimean war, with the enormous number of operations, not one life was lost; that ether is pungent, irritating to the lungs, and especially fatal to old people, and is used in America because it is an American invention.

In this country the feeling is overwhelmingly in favor of ether, on account of its greater safety.

1. Ether should not be administered to aged persons with emphysema, hypertrophy of the heart, fatty heart, or great valvular leisons.

2. It should not be administered to persons who faint easily.

3. It should not be administered to habitual drunkards, or those who drink a little each day.

4. It should not be administered in cases where the action of the lung is limited, either from old pleuritic adhesion or pneumonia, or whether there is an excessive secretion from the mucus membrane.

For the dentist's chair, nitrous oxide holds pre-eminence, on account of the quick return to consciousness; yet eight deaths from its use are already published. The same rule as to reclining posture and loose dress should be followed as to chloroform. It does not stimulate the sexual functions of both sexes; and the same precautions should also be observed to have third parties present.

One of the deaths from nitrous oxide was an English physician, and it was administered by his own dentist, who agreed to make him snore before commencing. The administrator should never take his direction from the patient, but use his own enlighted judgment as to what is best for that patient.

Numerous other anæsthetics have been brought forward for public favor, but they have not yet passed beyond the region of experiment.

Notwithstanding all the attention to the subject of anæsthetics, and all the study that has been made of this mystery of human life, it is not probable that we shall ever discover a perfectly safe and harmless way of parting with our sense of pain and be quite certain of a return to life. And while the number of deaths from anæsthetics are infinitesimal when compared with deaths from shock and unrelieved cases before anæsthetics were discovered; while anæsthetics have added greatly to the comfort of the patient; still the surgeon should remember every time he takes an anæsthetic in his hand he is leading the patient far down on the road to death, from which there may be no recovery.

Our fathers had to deal with men and women of more rugged constitutions than we have at the present day. The enervating effects of modern civilization had not reduced them to a bundle of nerves. There were no steam railways to give the terrific shock of collision, or machinery propelled by steam to crush and mangle. With all these increased difficulties over what our fathers had, the surgeon of the present day has to contend.

Statistics on a large scale show that with anæsthetics we produce as good results under these unfavorable conditions as our fathers did in the operations they had to meet. Who of us would go back to their iron-bound operating table, with holes through the irons to strap the patient down; to listen to the shrieks of the patient, and see the quivering limb almost baffling the surgeon's effort to sieze and secure the artery! Who would see the shock repeated on the nervous constitutions of to-day—the patient as then often dying from fear, or the rupture of an aneurism, or an apoplectic attack before the surgeon's knife had commenced its necessary work of torture!

To sum up, the medico-legal points made, are:

1. Anæsthetics do stimulate the sexual functions; the ano-genital region is the last to give up its sensitiveness. Charges made by females under the influence of an anæsthetic should be received as the testimony of an insane person is. It cannot be rejected; but the *corpus delicti aliunde* rule should be insisted on. Dentists or surgeons who do not protect themselves by having a third person present do not merit much sympathy.

2. Death from administration of chloroform after a felonious assault, unless the wounding was an inevitable fatal one, reduces the crime of the prisoner from murder to a felonious assault.

3. The surgeon has no right to use chloroform to detect crime against the will of the criminal.

4. The army surgeon has the right to use chloroform to detect malingerers.

5. The medical expert, notwithstanding he is sent by order of court, has no right to administer an anæsthetic against the wish of the plaintiff in a personal damage suit, to detect fraud.

6. Gross violations of the well-known rules of administering anæsthetics, life being lost thereby, will subject the violator to a trial on the charge of manslaughter.

7. A surgeon allowing an untrained medical student to administer anæsthetics, and life being thereby lost, will subject the surgeon himself to a suit for damages. What he does through his agent he does himself.

8. The physician who administers an anæsthetic should attend to that part of the work and nothing else. He should have carefully examined the heart and lungs beforehand. He should have the patient in the reclining position, with his clothes loose, so as not to interfere with respiration; should have his rat-tooth forceps, nitrate of amyl, and ammonia, and know their uses, and when to use them and artificial respiration.

9. In operations on the ano-genital region and the evulsion of the toe nail, complete loss of sensation in these parts should never be allowed, and no operation on these parts at all should be had under an anæsthetic unless by the approval of a full consultation, by those who have a knowledge of the dangers.

10. Chloroform cannot be administered to a person who is asleep without waking them, by a person who is not an expert. Experts themselves, with the utmost care, fail more often than they succeed in chloroforming adults in their sleep.

Another question I should have discussed should time have permitted, is whether a physician has the right to administer anæsthetics to mitigate death agonies. Take hydrophobia for instance, when death is inevitable; when the paroxysms of pain are frightful; when the danger to the surgeon in the administration in the ordinary way is extreme. Has he any right to alleviate this suffering, when the patient may pass away suddenly from the chloroform? A few years ago, a clergyman was convicted of murder in the second degree in England. He was a missionary among the poor in London, and when he found them with cancer and other incurable diseases, and without the means to obtain necessaries for their comfort, at the sick person's request he would administer a dose of morphia sufficient to carry them off, and he was transported for life as a convict for thus relieving incurable suffering. Would the physician who intentionally administered chloroform enough to a hydrophobic patient to cut short his suffering come under the same rule?

